# Comparing Farm Biosecurity and Antimicrobial Use in High-Antimicrobial-Consuming Broiler and Pig Farms in the Belgian–Dutch Border Region

**DOI:** 10.3389/fvets.2020.558455

**Published:** 2020-10-30

**Authors:** Nele Caekebeke, Franca J. Jonquiere, Moniek Ringenier, Tijs J. Tobias, Merel Postma, Angelique van den Hoogen, Manon A. M. Houben, Francisca C. Velkers, Nathalie Sleeckx, J. Arjan Stegeman, Jeroen Dewulf

**Affiliations:** ^1^Veterinary Epidemiology Unit, Department of Reproduction, Obstetrics and Herd Health, Faculty of Veterinary Medicine, Ghent University, Merelbeke, Belgium; ^2^Farm Animal Health Unit, Department of Population Health Sciences, Faculty of Veterinary Medicine, Utrecht University, Utrecht, Netherlands; ^3^GD Animal Health, Deventer, Netherlands; ^4^Experimental Poultry Centre, Geel, Belgium

**Keywords:** antimicrobial use (AMU), farm biosecurity, preventive measures, intensive livestock, alternatives to antimicrobials

## Abstract

As antimicrobial resistance is a worldwide problem, threatening both livestock and public health, understanding the drivers for resistance in different settings and countries is essential. Therefore, 30 pig and 30 poultry farms with country-specific high antimicrobial use (AMU) were recruited in the Belgian–Dutch border region. Information regarding production parameters, farm characteristics, biosecurity, and AMU was collected. On average, more biosecurity measures were implemented on Dutch farms, compared to Belgian farms in both animal species. In addition, more opportunities were found to increase the level of internal biosecurity compared to external biosecurity in both countries. AMU, quantified as treatment incidence (TI), differed marginally significant between broiler farms in Belgium and the Netherlands (median BE: 8; NL: 3), whereas in weaned piglets (median BE: 45 and NL: 14) and finishing pigs (median BE: 5 and NL: 1), there was a substantial difference in AMU between farms from both countries. Overall, Dutch farms showed less between-farm variation in TI than did Belgian farms. In both poultry and pig production, the majority of antimicrobials used were extended-spectrum penicillins (BE: 32 and 40%; NL: 40 and 24% for poultry and pigs, respectively). Compared to Belgian farms, Dutch poultry farms used high amounts of (fluoro)quinolones (1 and 15% of total AMU, respectively). None of the production parameters between broiler farms differed significantly, but in pig production, weaning age in Belgian farms (median: 23) was lower than in Dutch farms (median: 27). These results indicate considerable room for improvement in both countries and animal species. Farm-specific preventive strategies can contribute to lowering the risk for animal disease and hence the need for AMU.

## Introduction

It is estimated that, by 2050, antimicrobial resistance (AMR) could contribute to 10 million human fatalities per year worldwide if no actions are taken (1). The selection of AMR is largely driven by the (incorrect) use of antimicrobials (AM). As the development of new AM is limited (1, 2), treatment options are diminishing, endangering both human and animal healthcare.

The cross-border region of Belgium (Flanders) and the Netherlands is one with abundant movements of both humans and animals, due to high population numbers and intensive pig and poultry production, consequently posing a risk for dissemination of resistant bacteria and resistance genes, as AMR is not bound by country borders. Therefore, a multidisciplinary (One Health) approach needs to be complemented with cross-border cooperation to help understand and control the AMR problem (3).

AM in pig and poultry production are frequently administered orally, for group treatment of diseases of predominantly the respiratory and digestive tract (4–6). This method of treatment has a higher probability of improper dosing of the AM and contributes to the (over)exposure of healthy or non-infected animals to AM (7, 8). Therefore, these animal production systems need extra attention regarding their antimicrobial use (AMU).

Already in 2010, the Secretary of Agriculture of the Netherlands announced compulsory reductions of AMU in production animals (9, 10). This was quickly followed by a public–private program to reduce AMU in the Netherlands. In Belgium, AMU reduction plans were organized by the livestock sector (bottom-up approach) in 2012 and invigorated by the national government since 2016 to achieve the predetermined reduction goals. Substantial reductions in AMU have already been established in livestock in Belgium and the Netherlands (11, 12). Nevertheless, further reductions remain necessary, as high levels of AMR are still found (12–14).

By working together, both countries can learn from each other and harmonize methods of infection prevention, as it is believed that the latter will reduce the necessity for AMU, improving the safety of human and animal healthcare (15–17).

The objective of this manuscript is to describe and compare 30 pig and 30 poultry farms, selected for high AMU and located in the border region of Belgium and the Netherlands, with regard to production parameters, farm characteristics, biosecurity, and AMU. This inventory and comparison increase knowledge on potential associations between countries, species- or farm-specific parameters, and AMU, which can help identify where improvements should be made in order to reduce the problem of AMR.

## Materials and Methods

### Study Design and Data Collection

A cross-sectional survey was performed on 15 pig and 15 poultry farms in each country (60 farms in total). During a farm visit, farm characteristics and biosecurity levels were determined. In addition, data on technical performance and AMU were obtained, going back 1 year preceding the visit. The farm visits during which data were collected took place between September 2017 and April 2018.

To minimize observer bias, the execution of farm visits was restricted to two researchers/veterinarians (one for Belgium and one for the Netherlands), who were trained simultaneously and conducted 10 mutual farm visits to align methodologies.

Before enrolling, all participating farmers were informed on the aim and methodology of the study. All farmers signed an informed consent form for the collection, exchange, and publication of data. The Animal Welfare Body from Utrecht University was consulted and concluded that the study was exempt for an ethical evaluation, as the project did not include experimental procedures with animals according to EC/2010/63.

### Farm Selection

In each country, farms were recruited by sending out public announcements via different channels (newsletters, agricultural magazines, and professional contacts of the authors). The inclusion of farms was based on a “first come, first served” principle and the following criteria in order to obtain comparable farms in both countries: (1) for farm type, poultry farms needed to be conventional broiler farms (i.e., no organic production or slow-growing breeds), as conventional farms represent the majority of the farm systems in both countries, and pig farms needed to be a sow farm with weaned piglets present on the premises; (2) for farm location, all farms needed to be located within the Belgian–Dutch border region, comprising Flanders (northern region of Belgium) and the southern provinces of the Netherlands (Zeeland, Noord-Brabant, and Limburg); (3) for AMU, in the year preceding the farm visit, AMU needed to be above the national benchmark value, selecting for country-specific high users of AM. At the start of the project, no benchmark system was yet available for broilers in Belgium. Therefore, information provided by the farmer or herd veterinarian was considered. The latter criterion was included in view of further coaching the farmers toward a reduced AMU. To verify the inclusion of high-antimicrobial-consuming farms in this study, the AMU data retrieved from these farms were compared to national reference values (Supplementary Table 1).

Before or during the farm visits, one pig farm in Belgium and two broilers farms in the Netherlands withdrew from the project and were not replaced. Data of these farms are not presented.

### Farm Characteristics, Management, and Technical Performance

Farm characteristics and technical performance data were collected from the farmers in an interview and from farm management programs. The performance data were collected for 1 year preceding the farm visit for collection of the data. For all poultry farms, the number of houses with the total amount of broilers on the farm and the parameters mortality (during the 1st week and round total) and feed conversion ratio (FCR) were obtained for seven production rounds (about 1 year in total). At the pig farms, information regarding animal capacity (maximum amount of animals), weaning age, pre-weaning mortality, and the number of piglets per sow per year was collected.

### Biosecurity

The level of biosecurity was determined by completing the Biocheck.UGent™ questionnaire on-site in collaboration with the farmers and after visual appraisal of the farm. The questionnaire is a risk-based scoring system, evaluating the on-farm biosecurity in an objective manner (18, 19), resulting in a farm-specific report that scores external (all measures preventing the introduction of pathogens in the farm) and internal (all measures taken to prevent spread within the farm) biosecurity. The total biosecurity level on a farm is the weighted average of the external and internal biosecurity scores. Scores range from 0 to 100, with the latter being the implementation of all biosecurity measures, indicating the farmers' compliance to high biosecurity standards. Detailed information on the different subcategories within external and internal biosecurity can be found on the

$$\begin{array}{l} \text{TI}=\frac{\text{Total amount of active substance prescribed }\left(\text{mg}\right)}{\text{DDDvet }\left(\text{mg}/\text{kg}/\text{day }\right)\;*\;\left(\left(\text{observation period }*\text{ kg sow at risk}\right)+\;\left(\text{farrowing period }*\text{ kg sucklers at risk}\right)\right)}\;*\;100 \end{array}$$

website of Biocheck.UGent™ (https://www.biocheck.ugent.be) or in Gelaude et al. (19) and Laanen et al. (18).

To prevent interviewer bias, the Biocheck.UGent™ questionnaire was filled-in while or after doing a farm visit. This way, part of the answers to the questionnaire could be visually evaluated by the researcher.

### AMU Data

Data on AMU were obtained from the country-specific poultry or pork quality assurance organizations, the farmer, or the herd veterinarian. The data retrieved from either source are equal. However, collecting the data from the farmer or the veterinarian is much faster, as the reports provided by the quality organizations are only delivered a couple of times a year. To have data as soon as possible, the researchers got it directly from the farmer/veterinarian whenever possible.

The AMU was quantified in a standardized manner using the treatment incidence (TI) per 100 days as described by Persoons et al. (20) as the analysis of AMU data between Belgium and the Netherland differs in some aspects and as comparison is difficult without conversion. An overview of the different national monitoring systems is available on the AACTING website (https://www.aacting.org/monitoring-systems/).

The total amount of active substance prescribed equals the nominator, and the denominator represents the multiplication of (1) the defined daily dose (DDDvet, defined doses of an antimicrobial in mg per kg of animal), (2) the observation period (the number of days an animal is possibly exposed to a treatment), and (3) the amount of kg animals at risk. The ratio was then multiplied by 100 animal-days at risk to obtain the TI.

$$\begin{array}{l} \text{TI}=\;\frac{\text{Total amount of active substance prescribed }\left(\text{mg}\right)}{\text{DDDvet }\left(\text{mg}/\text{kg}/\text{day}\right)\;*\text{ observation period }*\text{kg animals at risk }}\;*100 \end{array}$$

The TI represents the percentage of time an animal was treated with AM during its life cycle.

For broilers, the observation period was the length of the production period. The kg animals at risk was determined by the standard weight for broilers of 1 kg corresponding with ESVAC guidelines (21), multiplied by the number of broilers on the farm. For weaners and finishers, the same formula was used, the standard weights of which, according to ESVAC, are 12 and 50 kg, respectively. However, the formula needed modification for sows and suckling piglets, as in Belgium, AMU in both categories is registered separately, whereas in the Netherlands, AMU in sows and suckling piglets are registered as one animal category. As disentanglement of the latter was not possible for data of the Dutch farms, an adjusted formula for AMU in sows and suckling piglets in both countries was determined:

The kg sow at risk is the multiplication of the standard weight of 220 kg for sows, according to ESVAC, with the number of sows present at the farm. Standard farrowing period was set at 28 days, the minimum weaning age according to EU legislation (EU Directive 2008/120/EEC). The value of kg sucklers at risk was calculated as the multiplication of the standard weight of suckling piglets (4 kg according to ESVAC), with a standard number of weaned piglets per sow per year of 28 (17), adjusted to the observation period and the number of sows: number of sows ^*^ (28/365 ^*^ observation period). An average number of piglets per sow per year was chosen to enable comparison between farms and countries.

The frequency of use of each antimicrobial class was determined and visualized by the number of prescriptions/total prescriptions. The antimicrobial classes were defined according to the ATCvet code (22).

### Data Analysis

Descriptive statistics were performed using IBM SPSS Statistics 25.0 (IBM, New York, United States). For comparison between countries and animal categories, the data are described by the mean value and the minimum-to-maximum range. Normality of the data was tested by visual inspection of the Q–Q plots. When data were not normally distributed, a logarithmic transformation of the data was performed. The equality of variances was tested by means of a Levene's test. An independent-samples *t*-test was used on all continuous variables whenever normality was demonstrated. Significance level was set at a *p* < 0.05. The 95% confidence interval (CI) for the difference in the mean was provided when significant differences were found. Results were rounded to whole numbers.

## Results

### Broiler Production

#### Farm Characteristics, Management, and Technical Performance

The main farm characteristics from the participating broiler farms are presented in Table 1. Most farms in Belgium had three (*n* = 6) to a maximum of four houses. In the Netherlands, most farms had two houses (*n* = 5), except for one farm with 10 houses. The total number of broilers per farm was higher in the Netherlands, with almost 117,000 broilers on average (median: 77,700) compared to just over 90,000 on average (median: 85,000) in Belgian farms. None of the production parameters differed significantly between both countries.

**Table 1:** Median and the minimum-to-maximum range of the most important characteristics of the participating broiler farms (Belgium *n* = 15, the Netherlands *n* = 13).

	**Belgium**	**Reference Belgium**	**the Netherlands**	**Reference the Netherlands**
Houses	3 [1–4]	NA	3 [1–10]	NA
Total broilers/farm	85,000 [50,000–180,000]	74,648^a^ (Flanders)	77,700 [23,400–490,000]	83,143^c^
Age of depopulation	41 [38–45]	42.4^a^ (Flanders)	41 [38–45]	41 [36–48]^d^
Mortality week 1 (%)	1.0 [0.3–2.9]	NA	1.0 [0.3–2.1]	NA
Mortality total (%)	2.9 [1.4–7.1]	3.3^a^ (Flanders)	3.0 [1.2–5.4]	3.5 [2.5–4.5]^c^^,^ ^d^
FCR total	1.6 [1.5–2.0]	1.61 [1.54–1.65]^a^^,^ ^b^	1.6 [1.5–1.7]	1.60 [1.33–1.65]^d^

*Data are from seven production rounds (± 1 year) preceding the farm visit. Median reference values for both countries were added*.

*FCR, feed conversion ratio*.

a *Department of Agriculture and Fisheries in Flanders (23);*

b *Pluimveeloket (24);*

c *Ago and food portal (25);*

d *Blanken et al. (26)*.

#### Biosecurity

Scores for biosecurity are represented in Table 2. Scores were on average significantly lower in the Belgian farms in comparison to those in the Dutch farms, for both internal and external biosecurity. From the different subcategories of external biosecurity, the best scoring subcategory in both Belgium and the Netherlands was infrastructure and biological vectors with, respectively, a median score of 78 and 93. This includes proper rodent control and prevention of direct contact between production animals and wild birds. One of the subcategories of the poultry questionnaire scoring low in both countries was feed and water supply, with 44 and 48 as median scores on Belgian and Dutch farms, respectively. All participating farmers yearly submitted water samples for quality analyses. However, 50% of the Dutch farmers took samples at the source, and the other half took samples both at the source and at the end of the line, with the latter being the ideal biosecurity measure. In Belgium, most farmers took only samples at the end of the waterline.

**Table 2:** Scores of the Biocheck.UGent™ questionnaire for broilers in the participating farms in Belgium (*n* = 15) and the Netherlands (*n* = 13).

		**Median score**	**Min–max score**
Belgium	External biosecurity	61	51–75
	Internal biosecurity	54	41–74
the Netherlands	External biosecurity	71	60–79
	Internal biosecurity	66	51–75

*The questionnaire was filled in during the farm visit, jointly by the external researcher and the farmer*.

Concerning internal biosecurity, house-specific and recognizable materials and farm clothing (subcategory materials and measures between compartments) were largely absent on farms in both countries. In four of the participating Belgian farms, there was an age difference between the flocks in different houses on the farm, with a maximum of 3 days, whereas broilers on participating Dutch farms were always of the same age across the houses. Detailed results per broiler farm are provided in Supplementary Table 2.

#### AMU

**Figure 1:**
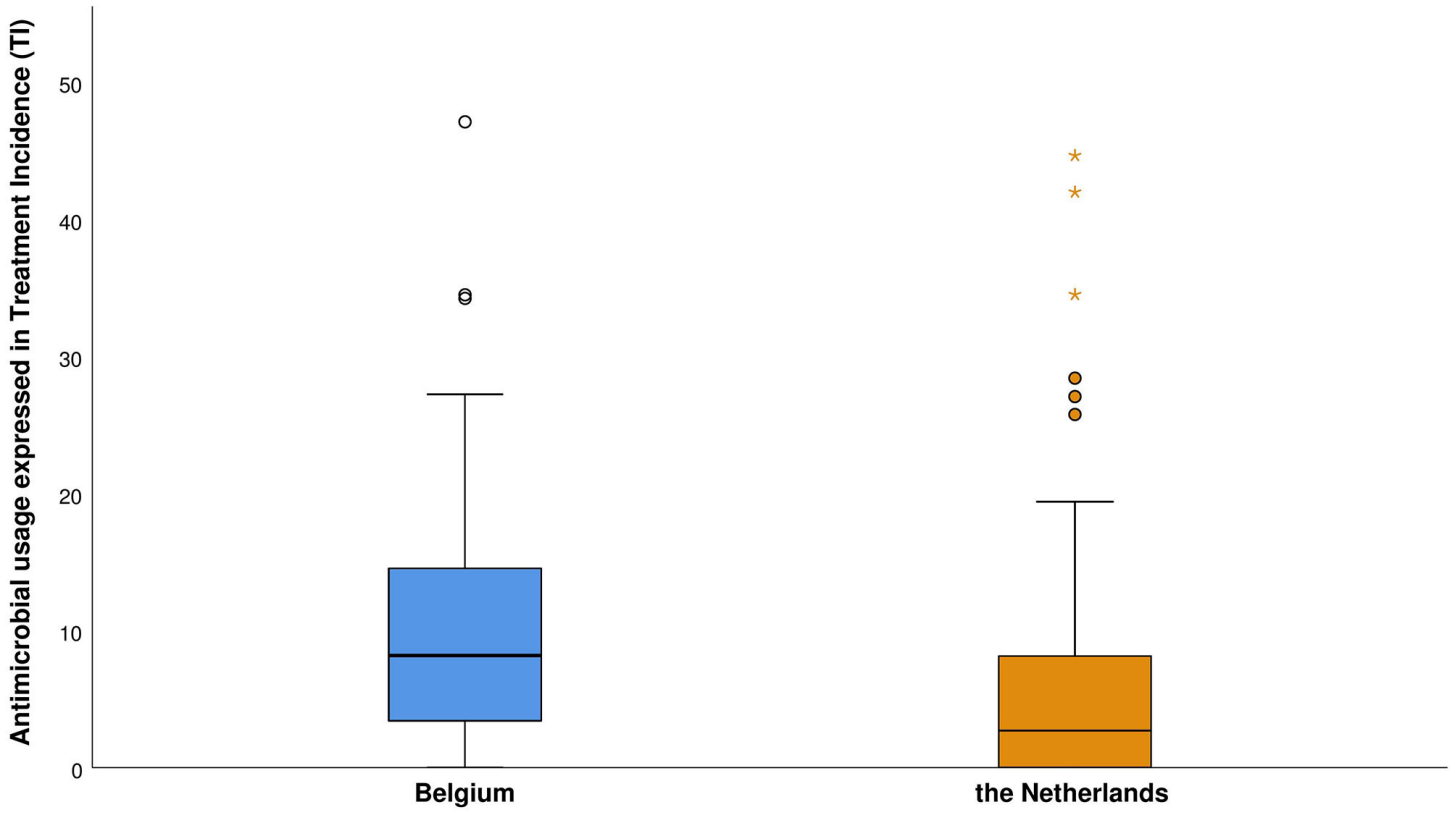
The antimicrobial use per production round of the participating broiler farms per country, based on seven rounds (± 1 year) preceding the farm visit. Antimicrobial use is expressed in treatment incidence (TI) on 100 days, i.e., the number of days an animal was treated with antimicrobials out of 100 days.

All of the AM applied on the poultry farms were administered via the drinking water. The median TI per production round per farm in Belgium, in the year before the farm visit took place, was 8 (range: 0–47, mean: 10). This equals treatment durations around 4 days on a standard production round of 42 days. On the Dutch farms, TI had a median value of 3 (range: 0–45, mean: 6). There was a marginally significant (*p*: 0.049) difference between the TI values in both countries, with a lot of variation between the different rounds within one farm and between farms per country (Figure 1). The AMU on farm level in each country is presented in the supplementary materials (Supplementary Figures 1, 2).

**Figure 2:**
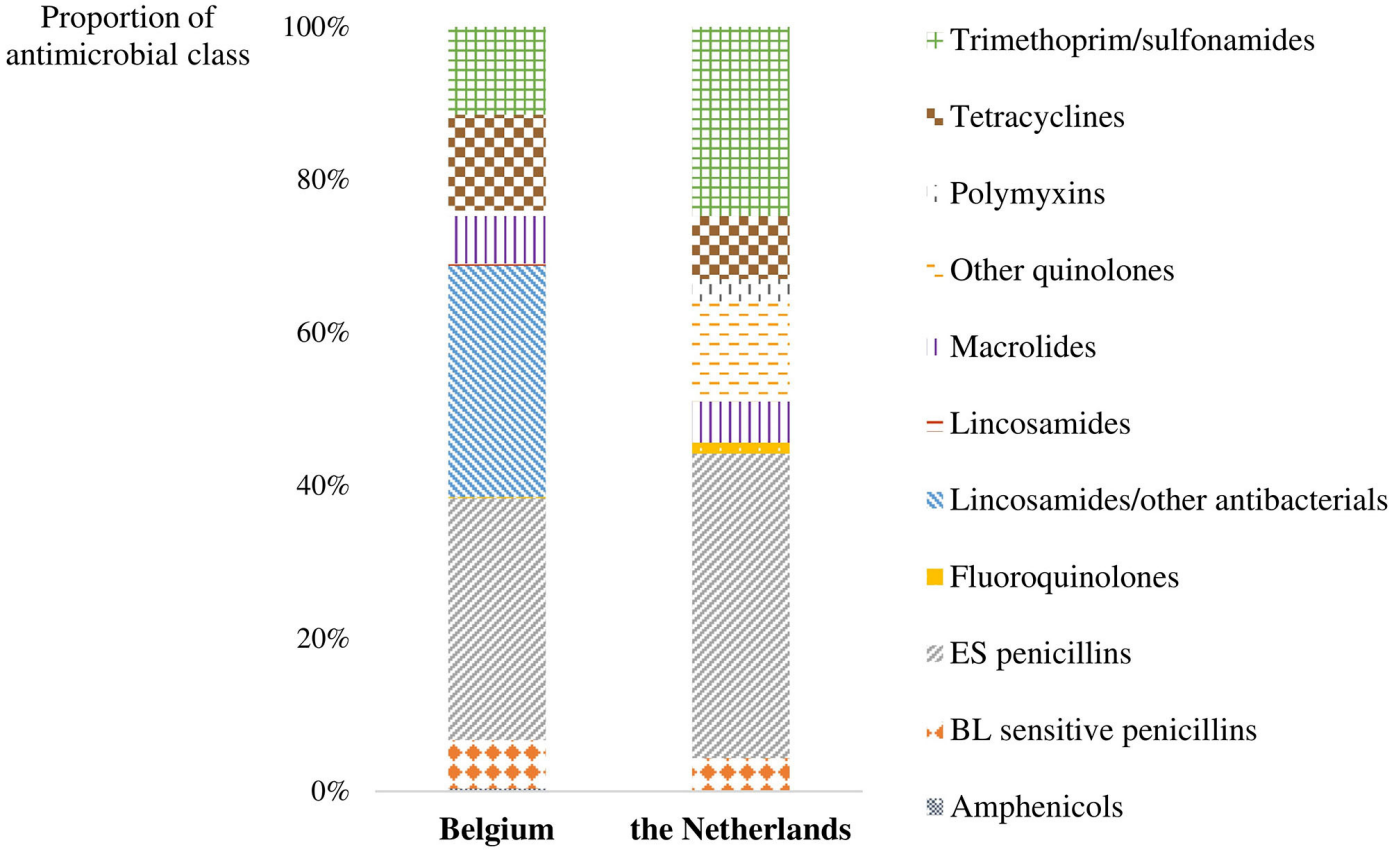
The proportion of each antimicrobial class prescribed on participating farms in Belgium and the Netherlands for broilers in the seven rounds preceding the farm visit. BL sensitive penicillins, β-lactamase sensitive penicillins; ES penicillins, extended-spectrum penicillins.

The majority of AM prescribed in broiler production were extended-spectrum (ES) penicillins (amoxicillin), with 32 and 40% of total registrations, respectively, for participating farms from Belgium and the Netherlands (Figure 2). In Belgian farms, just over 30% of AMU constituted a combination of lincomycin and spectinomycin, which was used on all farms. In the Dutch participating farms, 25% of total prescriptions constituted a combination of trimethoprim and a sulfonamide. However, this percentage is the result of the frequent use of the combination of trimethoprim and a sulfonamide on three Dutch farms.

(Fluoro)quinolones were used in < 1% of the prescriptions in Belgium. However, in the Dutch farms, this accounted for 15% of the total use. Colistin (antibiotic class of the polymyxins) was used on two Dutch farms, accounting for 3% of all prescriptions on the farms.

### Pig Production

#### Farm Characteristics, Management, and Technical Performance

The main production parameters from the participating pig farms are presented in Table 3. The average capacity on the Belgian farms was lower than on the Dutch participating farms for the different animal groups. Production in Belgium was organized in a 3- or 4-week batch farrowing system. In participating farms from the Netherlands, 1-week production systems were seen on the majority of farms (*n* = 10).

**Table 3:** Median and the minimum-to-maximum range of the most important production parameters of the participating pig farms (Belgium *n* = 14, the Netherlands *n* = 15).

	**Belgium**	**Reference Belgium**	**the Netherlands**	**Reference the Netherlands**
Capacity sows	326 [95–1,494]	233^a^	480 [315–1,600]	463^b^
Capacity weaners	1,238 [200–6,000]	NA	1,824 [800–8,000]	NA
Capacity finishers	2,143 [136–4,342]	1,465^a^	2,633 [300–14,350]	1,349^b^
Weaning age (days)	22.7 [19.3–30.8]	23.2^a^	26.7 [22.9–31.3]	23.4^d^
Mortality sucklers (%)	14.4 [2.4–24.7]	17^a^	13.0 [10.5–20.6]	14.2^d^
Weaned piglets/sow/year	30.9 [19.4–38.9]	25.6^a^	30.7 [26.7–33.5]	29.3^c^

*Data are from 1 year preceding the farm visit. Median reference values for both countries were added*.

*Capacity, maximum amount of animals that can be housed on the farm*.

a *Department of Agriculture and Fisheries in Flanders (23);*

b *CBS (27);*

c *Ago and food portal (25);*

d *Agrovision (28)*.

The weaning age on farms from Belgium was significantly lower than on farms from the Netherlands (−3 days, 95% CI: [−5; −1]). Pre-weaning mortality was slightly higher on average (not significant) on Belgian farms, and the number of weaned piglets per sow per year was similar in farms from both countries.

#### Biosecurity

The participating Dutch farms scored on average higher for external and internal biosecurity in comparison with the Belgian farms (Table 4). Detailed information per farm is provided in Supplementary Table 3.

**Table 4:** Scores of the Biocheck.UGent™ questionnaire for pigs in the participating farms from Belgium (*n* = 14) and the Netherlands (*n* = 15).

		**Median score**	**Min–max score**
Belgium	External biosecurity	59	47–74
	Internal biosecurity	46	24–72
the Netherlands	External biosecurity	74	61–84
	Internal biosecurity	73	45–92

*The questionnaire was filled in during the farm visit, jointly by the external researcher and the farmer*.

The subcategory with the lowest scores on average for both countries was feed, water, and equipment supply (median scores of 27 and 53 for Belgian and Dutch farms, respectively). The best scoring subcategory in Belgium was purchase of pigs and semen (median score of 88), as more than half of the farms did not purchase any animals. The best scoring subcategory in the Dutch farms was vermin and bird control (median score of 100); all Dutch farms stated to have little to no problems with vermin and control programs were established on all farms. Above that, no companion animals were allowed into the stables.

Concerning internal biosecurity, the subcategory measures between compartments, working lines, and equipment had a median score of only 32 (64 in the Netherlands) on participating Belgian farms. On farms from both countries, the farrowing and suckling period had low median scores of 36 and 50 in Belgium and the Netherlands, respectively, as many farmers from both countries transferred piglets between different sows later than 4 days post-farrowing. Also, during castration, only one blade was used, and/or the blades were not disinfected after each piglet.

#### AMU

**Figure 3:**
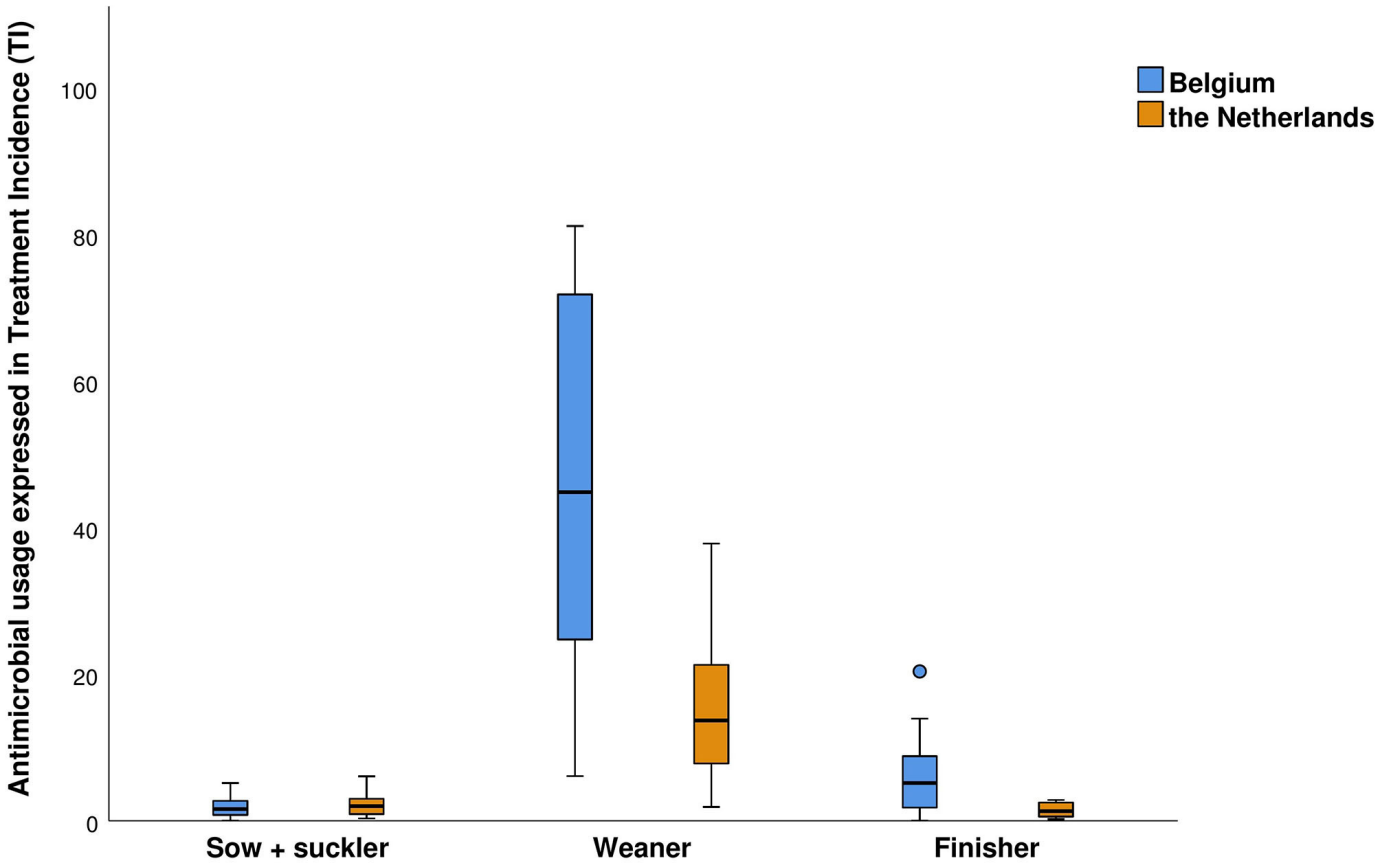
The antimicrobial use on the participating pig farms per country, 1 year preceding the farm visit. Antimicrobial use is expressed in treatment incidence (TI) on 100 days, which is the number of days an animal was treated with antimicrobials out of 100 days.

Variation was observed between different farms (Supplementary Figures 3–8), both within and between animal categories (Figure 3) and with respect to antimicrobial compounds used. For both countries, the majority of AMU was within the animal category of the weaners. In Belgium, AMU in the weaners ranged from a TI of 6 to over 80 (median: 45, mean: 46). In the finishers, AMU ranged from 0 to 20 (median: 5, mean: 6). AMU in the farrowing unit (sows + suckling piglets) was the lowest, ranging from 0 to 5 (median: 2, mean: 2). In the Dutch farms, overall AMU was lower and showed less variation in comparison to the Belgian participating farms. The AMU within the weaners ranged from 2 to 38 (median: 14, mean: 16) in the Dutch farms. The finishers showed the lowest average use with 0 to 3 (median: 1, mean: 1). Sows and their piglets had a TI of 0 to 6 (median: 2, mean: 2). There was a significant difference in TI of 30 within the weaners of each country [95% CI: (15; 45)] and a significant difference of 5 within the finishers of each country [95% CI: (1; 8)], where Belgian farms on average had a higher use.

**Figure 4:**
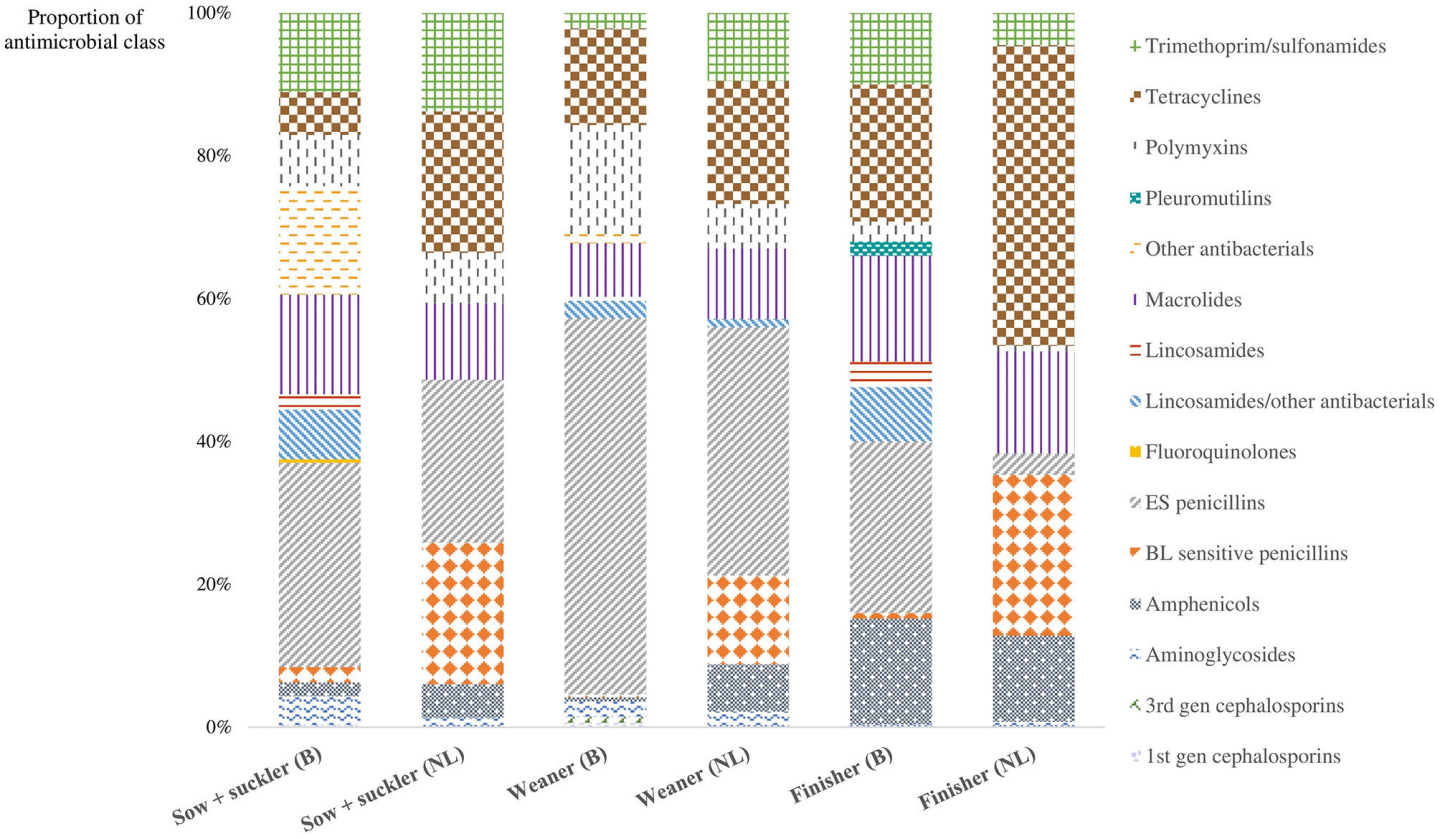
The proportion of each prescribed antimicrobial class in the Belgian and the Dutch farms for the different animal categories in pig production in the year preceding the farm visit. BL sensitive penicillins, β-lactamase sensitive penicillins; ES penicillins, extended-spectrum penicillins.

Figure 4 represents the proportion of the different antimicrobial compounds prescribed in all participating farms, per country. In Belgium, ES penicillin (amoxicillin and ampicillin) was the largest group of antimicrobial prescriptions in all animal categories, but especially in the weaners, where ES penicillins accounted for more than 50% of all prescriptions. In finishers, tetracyclines accounted for a large proportion of the prescriptions (19%) as well. The antimicrobial classes prescribed differed in the Dutch farms between different animal groups. In the weaners and the sows and suckling piglets, again the ES penicillins were prescribed the most (35 and 23%, respectively), whereas in the finishers, more than 42% of all prescriptions consisted of tetracyclines. Fluoro(quinolones) were used on two Belgian farms in the animal group of the sows and suckling piglets. One of those farms was also solely responsible for the proportion of third-generation cephalosporins in the Belgian weaners (1%). Polymyxins (colistin) were used in both countries in all animal categories, although the proportion was clearly larger in the Belgian compared to Dutch farms. The variety in antimicrobial classes prescribed was higher in the Belgian participating farms (15 vs. 9 classes in the Netherland).

## Discussion

This study has provided an inventory and comparison of poultry and pig farms with high AMU in Belgium and the Netherlands with regard to farm-specific performance, management, biosecurity, and AMU in order to identify opportunities for improvements. Overall, Dutch farms scored better on biosecurity, but internal biosecurity needs more attention in both countries. The Dutch farms had a lower AMU in broiler farms in comparison to Belgian farms; however, a higher amount of critically important AM for use in human medicine was used. In pig production, the AMU was significantly higher within the weaners and finishers of the Belgian farms.

Production parameters from the participating farms were similar in both Belgium and the Netherlands, except for weaning age in pig production. As 3- to 4-week batch farrowing systems occurred more frequently in the Belgian farms, the average weaning age was expected to be lower in comparison to the Dutch participating farms, where most farms worked with a 1-week production system. However, the higher weaning age in Dutch farms and a significantly lower AMU within the weaners are supporting previous findings that weaning at an earlier age may have a negative effect on AMU (15, 29).

With regard to biosecurity, the low scores for the subcategory feed and water supply for both countries and animal species were remarkable. These low scores were mainly linked to questions with regard to water quality, emphasizing the need for more attention to the importance of good-quality drinking water for animal health. The results from this study also suggest that there is more room for improvement in the measures linked to internal biosecurity compared to external biosecurity measures. Both findings are in line with the national biosecurity data as presented on the Biocheck.UGent™ website (30). The lower scores for internal biosecurity could be explained by a bigger awareness of the farmers for the risk of introduction of disease coming from other farms (31) or the belief that it is easier to impose guidelines upon external visitors than to change habits on the farm (18).

In both broiler and pig production, the average biosecurity levels from the Dutch participating farms were higher than those on the Belgian farms. This is in accordance with previous studies, where Belgian farms did not score very high on their biosecurity level (19, 31–34). The establishment of reduction goals for AMU already in 2010 (6 years earlier than government-supported goals in Belgium) could have encouraged the Netherlands to increase biosecurity on animal farms sooner in order to keep their animals safe.

The earlier initiation of reduction goals by the Dutch government also shows its positive effects in AMU reduction numbers in comparison to Belgium on a national level (4). This earlier adaptation of reduction goals in the Netherlands may be linked to high public pressure on AMU reduction in the Netherlands as a result of the discovery at that time that production animals could be reservoirs for antimicrobial resistant bacteria (e.g., methicillin-resistant *Staphylococcus aureus*) (10). The effect of these measures can explain the overall lower AMU in the Dutch participating farms in comparison to the Belgian farms.

The lower level of AMU in the Dutch broiler farms was partially the result of multiple production rounds where no AM were used. This illustrates that it is possible to raise animals without the use of AM. Therefore, there is still a lot of room for reducing AMU in high-consuming farms.

There was a big difference in AMU in pig production between both countries. However, we did not find significant differences in production parameters, except for weaning age, suggesting that room for improvement is possible in Belgium, without risking negative effects on technical performances (16, 17, 35, 36). The overall lower AMU in the Netherlands could also be a consequence of the overall higher biosecurity levels, as biosecurity as an alternative to AMU was already described in previous studies (15, 37, 38).

Due to the selection criteria, it should be noted that the farms included in this study cannot be considered representative for the full pig and broiler production in Belgium and the Netherlands. For this study, only farms within certain geographical borders were selected, and the selected farms had an AMU above the national benchmark value. In addition, the selection on a first come, first served basis may have led to a sample of farmers with a preexisting interest in AMU/AMR reduction. Moreover, as 30 farms per animal species were enrolled in this study, we only included a small part of the entire production in both countries. However, due to this limited number, detailed information per farm could be obtained.

Calculation and expression of AMU in both countries differed, which made recalculations necessary before comparison between countries was possible. As two different formulas were required for AMU calculation in pig production, no TI covering the entire lifetime of a pig could be calculated. Therefore, no total AMU in pig production could be measured and consequently compared to broiler production. These differences highlight the need for European harmonization in calculating AMU if a valid comparison between countries is aimed for.

The differences in policy regarding AMU between Belgium and the Netherlands might explain the different uses of antimicrobial classes between both countries in this study. For instance, the definitions of first- and second-choice compounds differ sometimes per country; e.g., in Belgium, macrolides are always considered second choice in pigs and poultry (39), whereas in the Netherlands, depending on the active compound and animal species, macrolides can be first or second choice (40). The same applies to quinolones, which are second-choice compounds in the Netherlands and third choice in Belgium. These differences raise questions, as the effect of AM on the bacteria is the same, regardless of the country. Especially concerning the critically important AM used in human medicine (41), again, some harmonization would be welcomed here as these different classifications often cause confusion among farmers and veterinarians working in a border region.

In both the Netherlands and Belgium, there is a ban on the preventive use of AM, and the combination product lincomycin with spectinomycin is classified as second choice in broiler production. Broiler farmers have used this product often in the 1st days post-hatch to reduce the risk of bacterial chondronecrosis and osteomyelitis of the femoral head due to *Enterococcus cecorum* later in the production period, which is associated with severe locomotion problems and high therapeutic use of AM with limited success (42). The absence of use of this combination product in the Dutch farms, in comparison to its high use in the Belgian farms, can be explained by the strict repercussions for farmers or veterinarians in the Netherlands due to not following legislation, which is not yet present in Belgium.

No cephalosporins or (fluoro)quinolones, belonging to the critically important AM for use in human medicine, were applied in pig production in the Netherlands in the year preceding the farm visit. This shows that it is possible to rear animals without using these AM.

## Conclusion

In this paper, we used a standardized methodology for collection and analysis of the data in two countries and animal species, making it possible to compare participating farms with respect to farm characteristics, biosecurity, and AMU.

Important differences in AMU between both countries were found. The higher weaning age in Dutch pig production, associated with a lower AMU, could indicate the benefits of higher weaning age on AMU, especially as most AM were used within the weaners. The use of critically important AM for human medicine in livestock production should be further investigated to limit this use as much as possible.

Reduction targets for AMU on a national level can drive the reduction of AMU on farm level supported by many different management, housing, and feeding measures, among which improved biosecurity is certainly an important component. Further investigation into the specific preventive measures that could offer the biggest benefits for AMU reduction is needed. To improve sustainability and compliance of these measures, change management techniques may prove useful (43). The farms in this study will be followed up for 1 year, where improvement in biosecurity will be the main target in combination with the coaching of the farmers toward increased animal health.

## Data Availability Statement

The datasets presented in this article are not readily available because the project management needs to give it's approval whether databases can be shared. Requests to access the datasets should be directed to i41health@amphia.nl.

## Ethics Statement

Before enrolling, all participating farmers were informed on the aim and methodology of the study. All farmers signed an informed consent form for the collection, exchange and publication of data. The Animal Welfare Body from Utrecht University was consulted, and concluded that the study was exempt for an ethical evaluation, as the project did not include experimental procedures with animals according to EC/2010/63.

## Author Contributions

NC, MR, TT, MP, AH, MH, FV, NS, JS, and JD contributed to conception and design of the study. NC, MR, and AH (afterwards replaced by FJ) performed the farm visits and collected all data. NC, MR, and FJ organized the database. NC performed the statistical analysis and the writing of the manuscript. NC, MR, TT, MP, FV, NS, JS, and JD contributed to manuscript revision, read, and adjustments. All authors approved the submitted version.

## Conflict of Interest

The authors declare that the research was conducted in the absence of any commercial or financial relationships that could be construed as a potential conflict of interest.
