# Supplementary material for: Comparing Farm Biosecurity and Antimicrobial Use in High-Antimicrobial-Consuming Broiler and Pig Farms in the Belgian–Dutch Border Region
Source: Front Vet Sci. 2020 Oct 30;7:558455. doi: 10.3389/fvets.2020.558455 (PMC7673451; doi:10.3389/fvets.2020.558455)
Supplement: Supplementary file 1 [file Data_Sheet_1.pdf]

Supplementary Material

Supplementary Table 1 Antimicrobial use data from the performed study (expressed in Treatment Incidence, TI) with reference values per country for the different animal categories (2018). A comparison within the category of the sows and suckling piglets was not possible due to major adjustments in the calculation of antimicrobial use for this animal category in the manuscript, which made comparison impossible. This table indicates that we have selected high antimicrobial consuming farms in this study.

		**Study mean**	***Reference mean***	**Study median**	***Reference median***
**Broilers**	Belgium[1]	10	*7.2*	8	*5.4*
	the Netherlands[2]	6	*3.9*	3	*2.8*
**Weaners**	Belgium[1]	46	*27.5*	45	*16.6*
	the Netherlands[2]	16	*5.4*	14	*2.8*
**Finishers**	Belgium[1]	6	*4.4*	5	*2.8*
	the Netherlands[2]	1	*1.1*	1	*0.5*

[1]Reference values for Belgium were retrieved from the national data collection system Sanitel-Med, with calculations done by AMCRA (Centre of expertise on antimicrobial use and resistance in animals) (38).
[2]Reference data for the Netherlands was retrieved from the SDa's (Netherlands Veterinary Medicines Institute) annual report on "usage of antibiotics in agricultural livestock in the Netherlands in 2018" (39) and recalculated to 100 days.

Supplementary Table 2 Biosecurity scores (0 to 100) of the participating broiler farms for the Netherlands (n=13) and Belgium (n=15) after completion of the Biocheck.UGent™ questionnaire. The higher the score, the more biosecurity measures were at place.

	**the Netherlands**													**Belgium**														
Farmnumber	*1*	*2*	*3*	*4*	*5*	*6*	*7*	*8*	*9*	*11*	*12*	*14*	*15*	*1*	*2*	*3*	*4*	*5*	*6*	*7*	*8*	*9*	*10*	*11*	*12*	*13*	*14*	*15*
**Total external biosecurity**	**75**	**66**	**63**	**66**	**62**	**60**	**66**	**79**	**77**	**77**	**78**	**76**	**71**	**57**	**67**	**63**	**65**	**51**	**56**	**59**	**59**	**61**	**67**	**67**	**75**	**57**	**52**	**68**
Purchase one-day-old chicks	79	79	79	48	30	59	79	58	79	79	79	79	59	79	90	79	79	9	79	58	79	79	79	79	79	37	30	79
Depopulation broilers	78	71	51	71	51	65	71	71	64	65	71	71	77	51	59	71	51	61	44	44	61	51	71	71	71	64	15	64
Feed and water	67	48	48	62	66	48	48	87	87	43	47	43	48	33	47	48	44	44	48	48	44	44	44	44	44	36	44	48
Removal manure & carcasses	73	66	88	77	66	78	53	88	88	88	88	88	77	53	63	53	52	48	63	53	77	62	77	63	63	77	63	66
Visitors and farmworkers	70	59	61	76	76	67	76	90	90	96	96	96	90	47	70	56	84	64	47	56	76	56	70	61	90	56	64	70
Material supply	100	44	44	56	56	0	56	56	56	56	56	56	56	44	56	44	56	56	44	44	0	56	56	56	100	44	56	56
Infrastructure and biological vectors	89	97	93	87	65	93	94	94	90	97	94	93	82	80	79	90	69	65	77	94	72	76	81	76	83	58	78	86
Location farm	42	48	30	30	81	44	30	81	48	81	81	67	63	63	63	44	81	44	44	63	44	63	44	81	63	81	67	63
**Total internal biosecurity**	**75**	**69**	**61**	**66**	**51**	**63**	**55**	**75**	**68**	**65**	**73**	**73**	**65**	**54**	**74**	**54**	**57**	**41**	**51**	**43**	**55**	**45**	**45**	**63**	**62**	**74**	**42**	**57**
Disease management	79	60	60	79	81	81	79	79	81	81	81	81	81	56	60	58	79	79	56	37	60	60	60	77	79	79	79	79
Cleaning and disinfection	59	55	50	62	55	68	37	68	67	63	62	63	58	65	70	65	55	18	58	54	48	41	41	71	50	66	35	38
Materials and measures btw compartments	100	82	82	53	0	29	53	82	53	53	82	82	53	29	100	29	29	29	29	29	59	29	29	29	59	82	0	59
**Total overall biosecurity**	**75**	**67**	**62**	**66**	**58**	**61**	**63**	**78**	**74**	**73**	**77**	**75**	**69**	**56**	**69**	**60**	**63**	**48**	**54**	**54**	**58**	**56**	**60**	**66**	**71**	**62**	**49**	**64**

Broiler farms 10 and 13 in the Netherlands withdrew from the project and were not replaced.

Supplementary Table 3 Biosecurity scores (0 to 100) of the participating pig farms for the Netherlands (n=15) and Belgium (n=14) after completion of the Biocheck.UGent™ questionnaire. The higher the score, the more biosecurity measures are at place.

	**the Netherlands**															**Belgium**													
Farmnumber	*1*	*2*	*3*	*4*	*5*	*6*	*7*	*8*	*9*	*10*	*11*	*12*	*13*	*14*	*15*	*1*	*2*	*3*	*4*	*5*	*6*	*7*	*8*	*9*	*10*	*11*	*12*	*14*	*15*
**Total external biosecurity**	**71**	**75**	**71**	**82**	**61**	**78**	**73**	**78**	**81**	**67**	**73**	**84**	**74**	**80**	**64**	**64**	**64**	**61**	**68**	**54**	**59**	**58**	**74**	**59**	**65**	**52**	**47**	**54**	**56**
Purchase pigs and semen	88	88	76	88	48	88	88	88	84	82	74	88	78	88	82	76	74	56	88	88	88	100	86	88	88	88	76	56	88
Feed, water and equipment supply	37	53	53	43	43	53	47	53	53	37	37	53	63	73	43	53	37	27	43	17	10	27	53	27	17	17	17	27	37
Removal animals, manure, carcasses	72	52	74	83	83	74	83	78	83	52	78	83	87	83	43	74	70	83	78	54	67	74	63	63	83	39	46	70	39
Visitors and farmworkers	88	76	88	88	76	88	100	88	88	76	76	88	88	88	53	59	65	47	65	53	53	35	82	47	53	35	35	47	65
Vermin and bird control	100	100	100	100	70	60	80	100	80	100	100	100	100	100	100	40	80	60	70	50	60	40	90	60	60	60	50	50	50
Location farm	20	100	20	100	40	100	0	50	100	60	80	100	0	30	80	60	50	100	30	30	50	20	70	40	70	60	40	70	30
**Total internal biosecurity**	**56**	**75**	**78**	**73**	**45**	**52**	**92**	**69**	**77**	**67**	**56**	**79**	**87**	**90**	**62**	**60**	**54**	**29**	**72**	**42**	**38**	**51**	**58**	**54**	**47**	**41**	**24**	**45**	**35**
Disease management	80	80	100	40	80	40	100	40	100	80	80	100	60	80	80	20	20	20	20	20	40	40	100	60	40	40	40	40	40
Cleaning and disinfection	30	85	60	98	50	40	85	95	95	85	65	95	95	95	85	65	75	30	75	65	10	50	30	45	10	40	20	50	20
Measures btw compartments, working lines and equipment	57	61	82	100	61	36	100	61	96	86	61	64	89	96	32	54	46	29	93	21	29	32	61	36	36	11	7	32	21
Farrowing and suckling period	29	50	50	43	14	64	64	50	21	36	36	50	86	86	71	64	21	43	14	36	29	36	36	36	64	71	29	21	71
Nursery unit	64	86	86	50	36	64	100	64	50	64	36	86	86	100	64	71	57	21	100	71	50	71	86	100	64	36	50	71	43
Finishing unit	93	100	100	57	21	86	100	93	NA	29	NA	93	93	71	NA	79	93	NA	93	NA	93	93	57	71	93	79	21	64	36
**Total overall biosecurity**	**64**	**75**	**75**	**78**	**53**	**65**	**83**	**74**	**79**	**67**	**65**	**82**	**81**	**85**	**63**	**62**	**59**	**45**	**70**	**48**	**49**	**55**	**66**	**57**	**56**	**47**	**36**	**50**	**46**

Pig farm 13 in Belgium withdrew from the project and was not replaced.
NA = not applicable

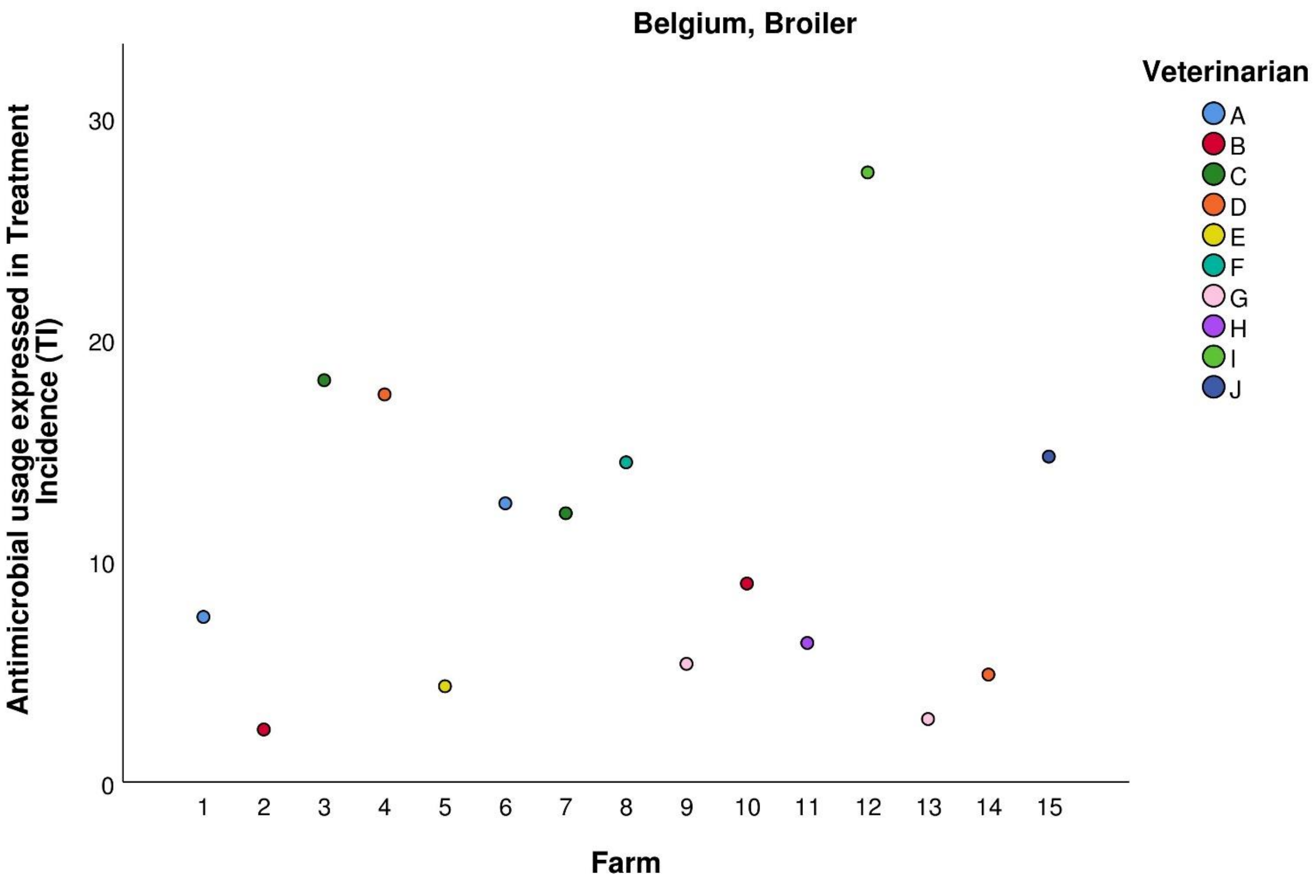

Supplementary Figure 1 Antimicrobial use of each participating broiler farms in Belgium (n=15). Antimicrobial use is expressed in Treatment Incidence (TI) on 100 days, the number of days an animal was treated with antimicrobials out of 100 days. The different colors represent the different veterinarians involved in this study.

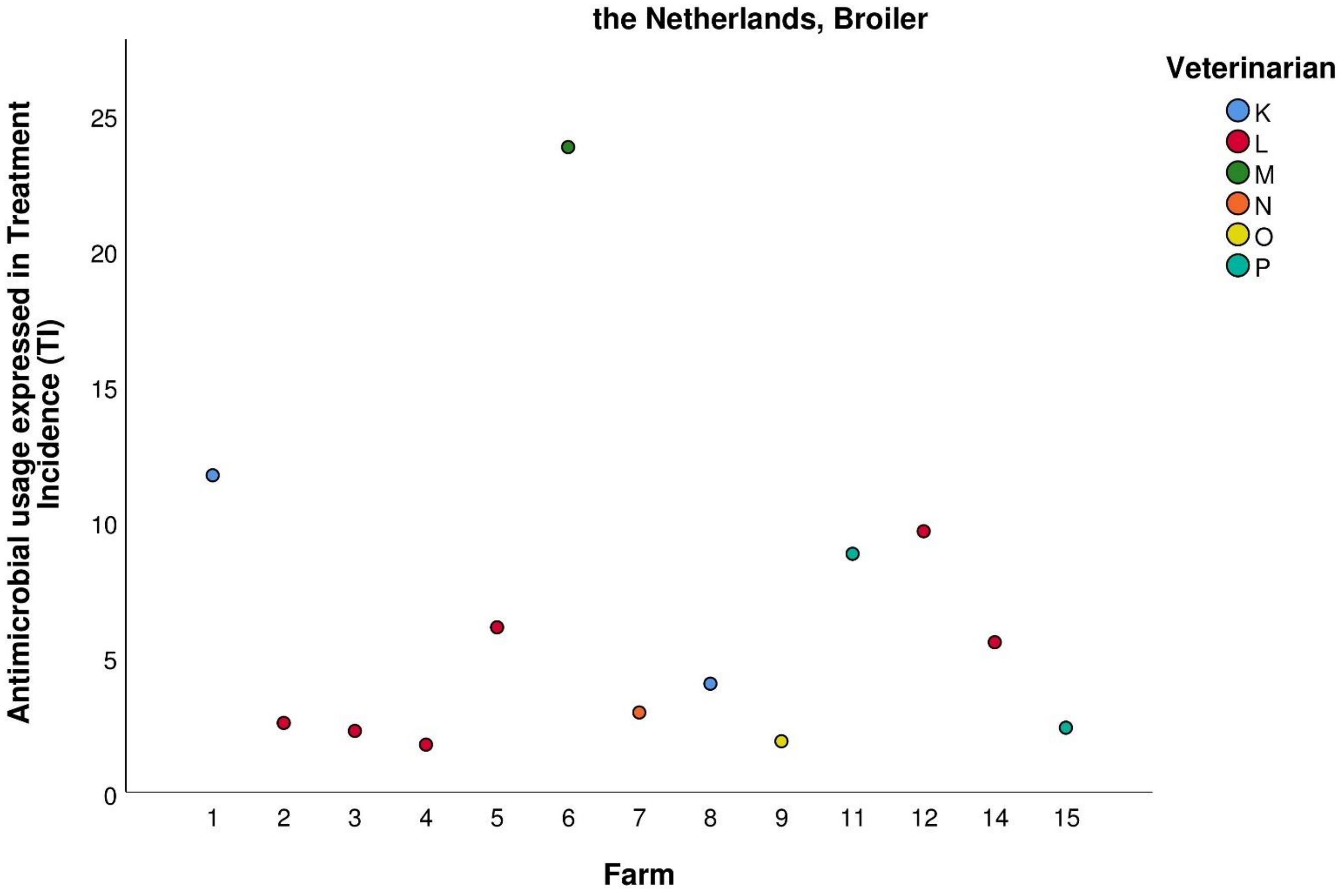

Supplementary Figure 2 Antimicrobial use of each participating broiler farms in the Netherlands (n=13). Antimicrobial use is expressed in Treatment Incidence (TI) on 100 days, the number of days an animal was treated with antimicrobials out of 100 days. The different colors represent the different veterinarians involved in this study. Farms 10 and 13 withdrew from the project and were not replaced.

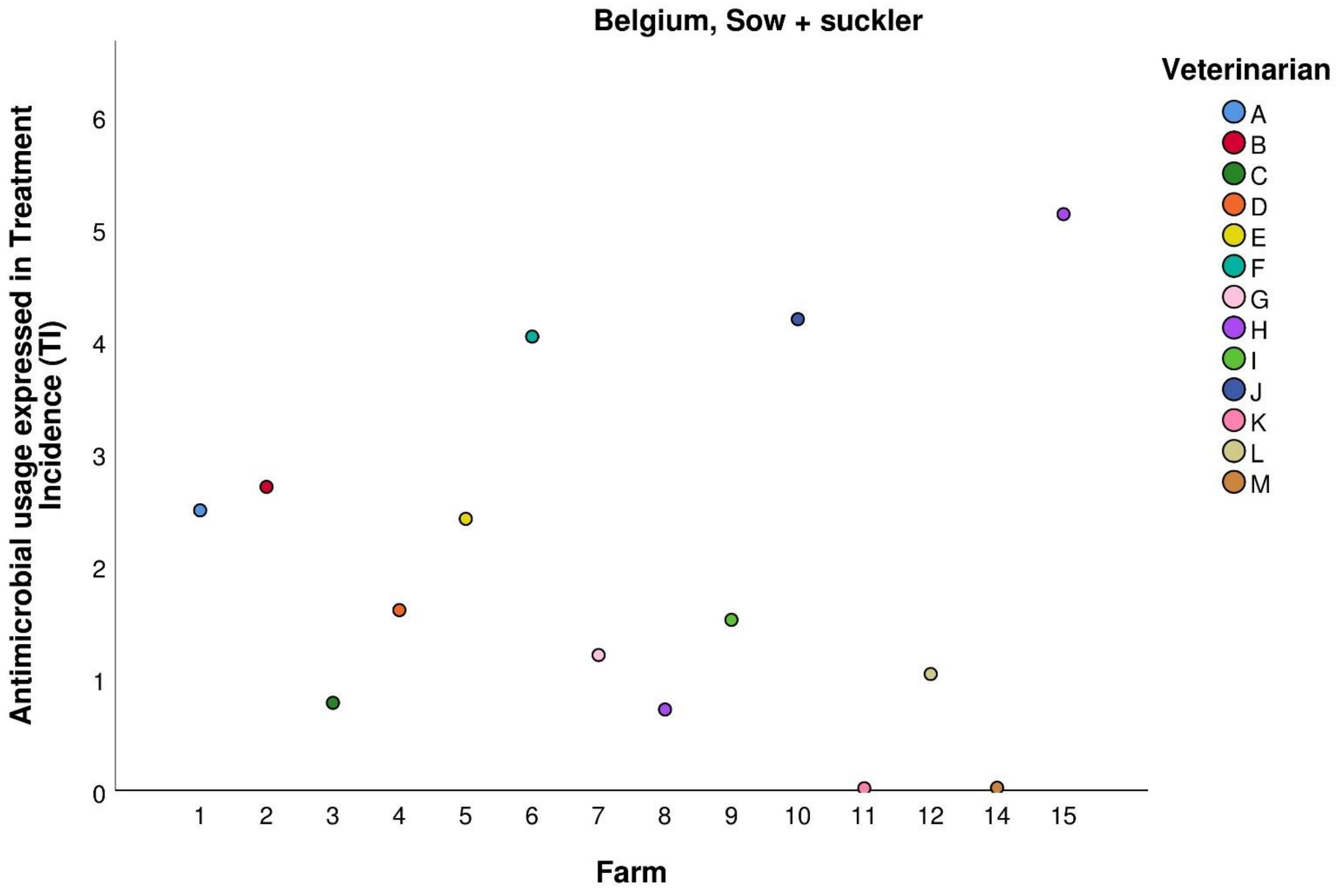

Supplementary Figure 3 Antimicrobial use of each participating pig farm in Belgium (n=14) for the animal category of the sows and suckling piglets. Antimicrobial use is expressed in Treatment Incidence (TI) on 100 days, the number of days an animal was treated with antimicrobials out of 100 days. The different colors represent the different veterinarians involved in this study. Farm 13 withdrew from the project and was not replaced.

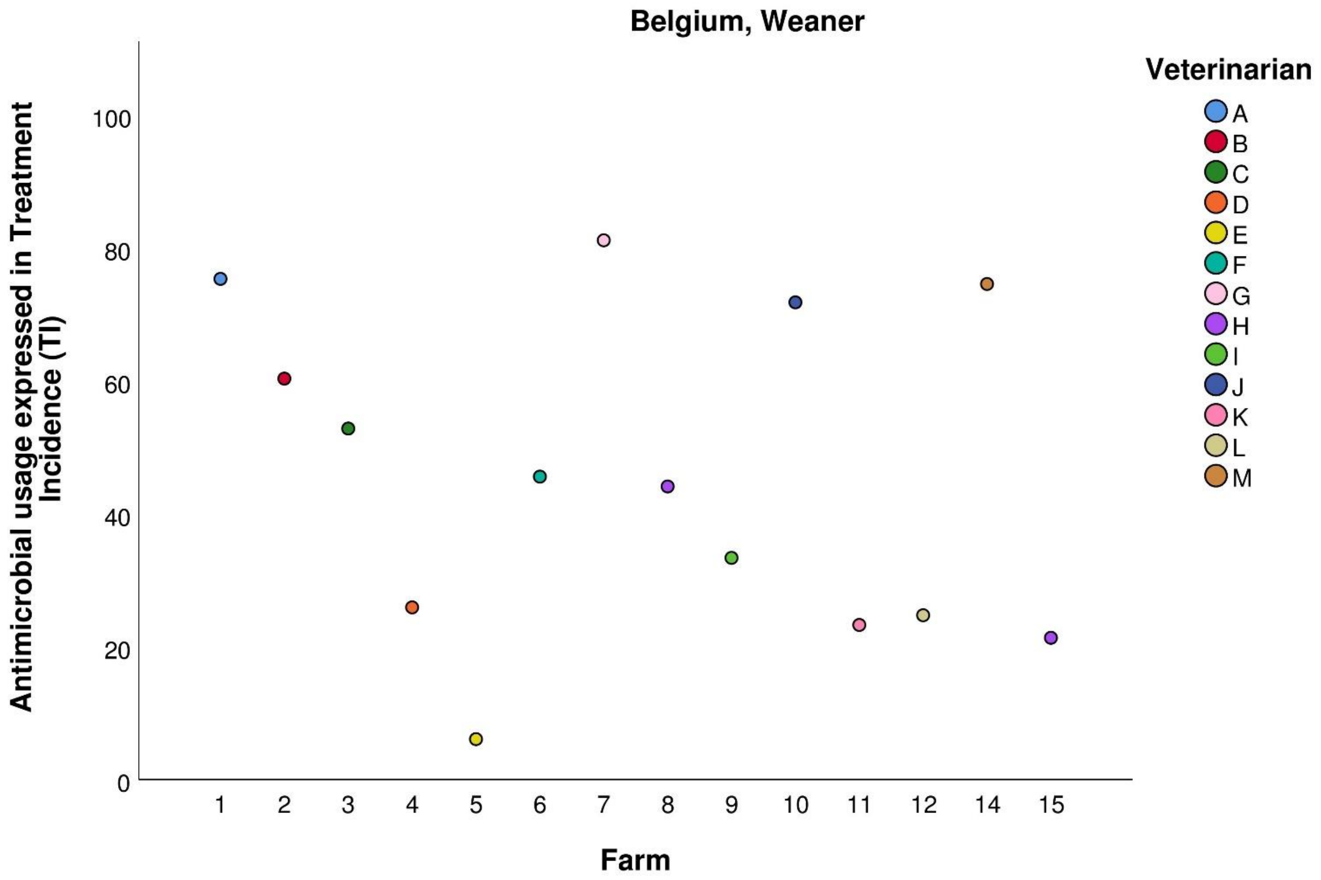

Supplementary Figure 4 Antimicrobial use of each participating pig farm in Belgium (n=14) for the animal category of the weaners. Antimicrobial use is expressed in Treatment Incidence (TI) on 100 days, the number of days an animal was treated with antimicrobials out of 100 days. The different colors represent the different veterinarians involved in this study. Farm 13 withdrew from the project and was not replaced.

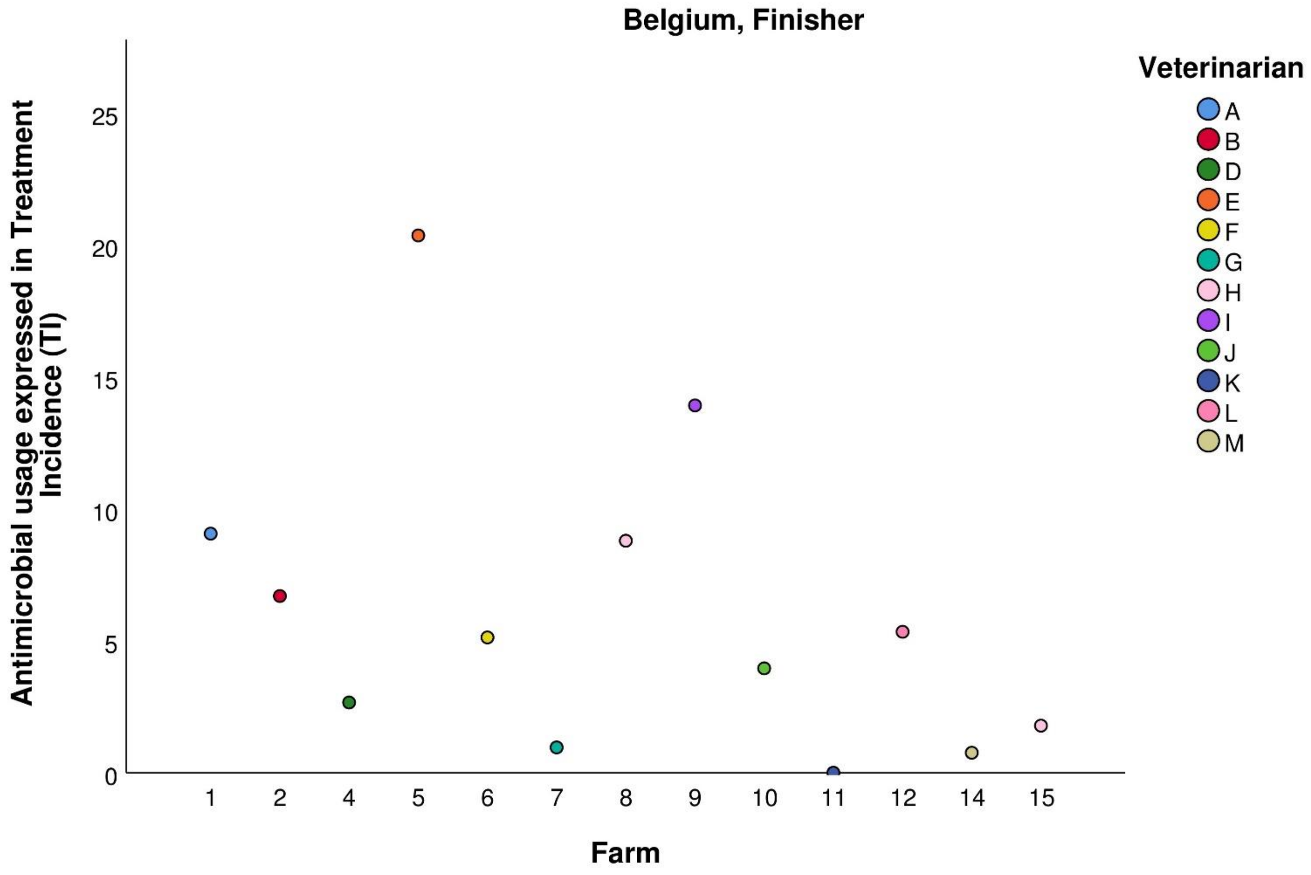

Supplementary Figure 5 Antimicrobial use of each participating pig farm in Belgium (n=14) for the animal category of the finishers. Antimicrobial use is expressed in Treatment Incidence (TI) on 100 days, the number of days an animal was treated with antimicrobials out of 100 days. The different colors represent the different veterinarians involved in this study. Farm 13 withdrew from the project and was not replaced. Farm 3 had no finishers.

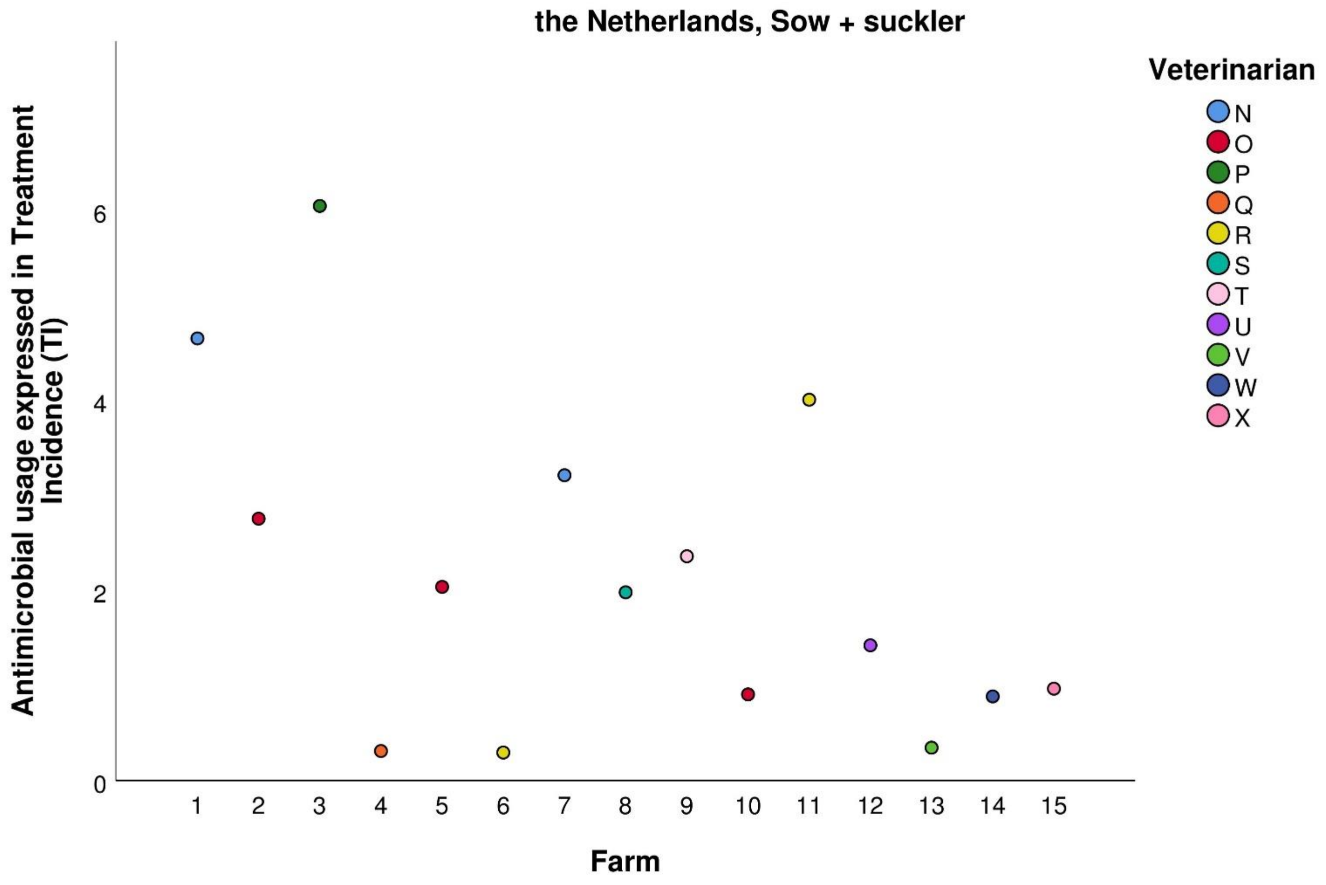

Supplementary Figure 6 Antimicrobial use of each participating pig farm in the Netherlands (n=15) for the animal category of the sows and suckling piglets. Antimicrobial use is expressed in Treatment Incidence (TI) on 100 days, the number of days an animal was treated with antimicrobials out of 100 days. The different colors represent the different veterinarians involved in this study.

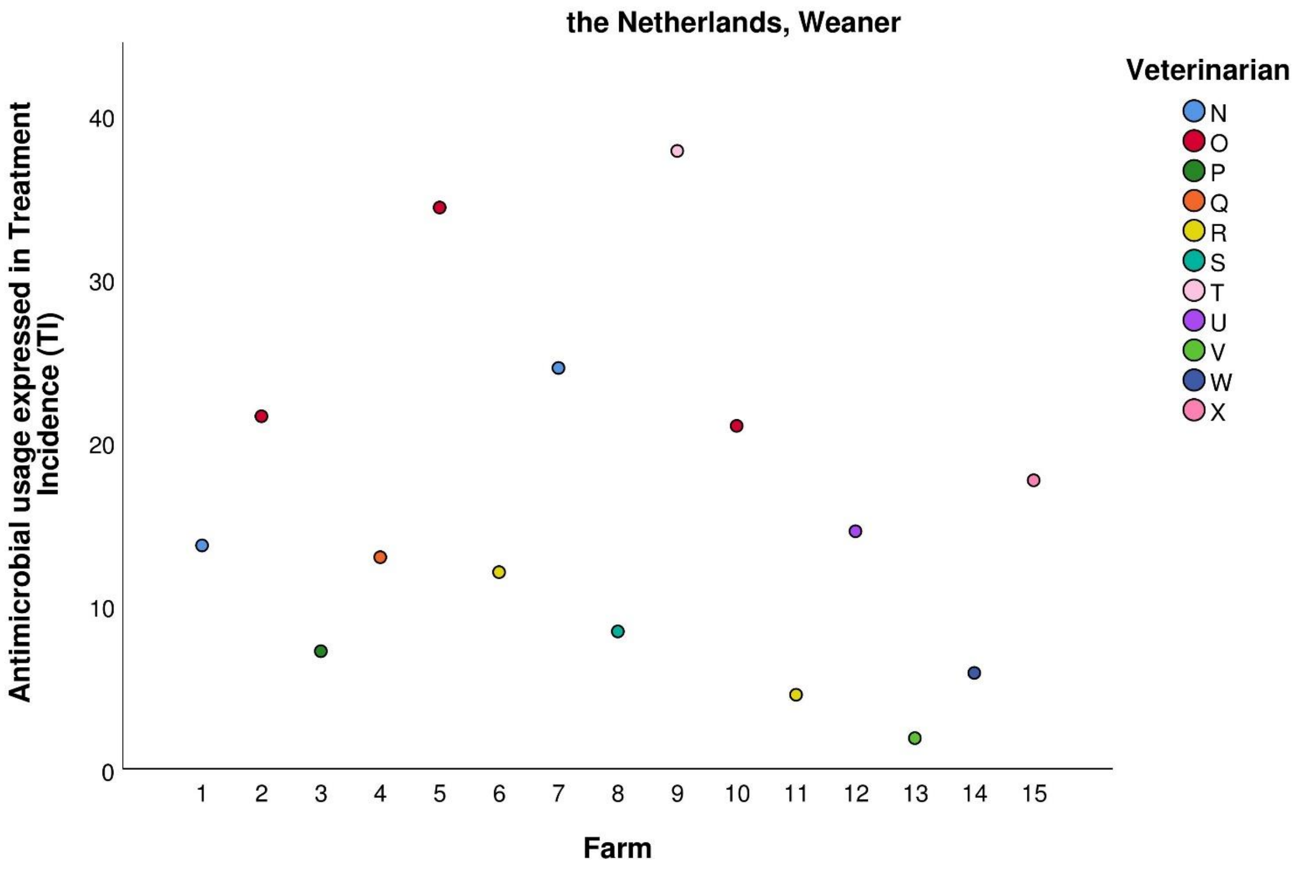

Supplementary Figure 7 Antimicrobial use of each participating pig farm in the Netherlands (n=15) for the animal category of the weaners. Antimicrobial use is expressed in Treatment Incidence (TI) on 100 days, the number of days an animal was treated with antimicrobials out of 100 days. The different colors represent the different veterinarians involved in this study.

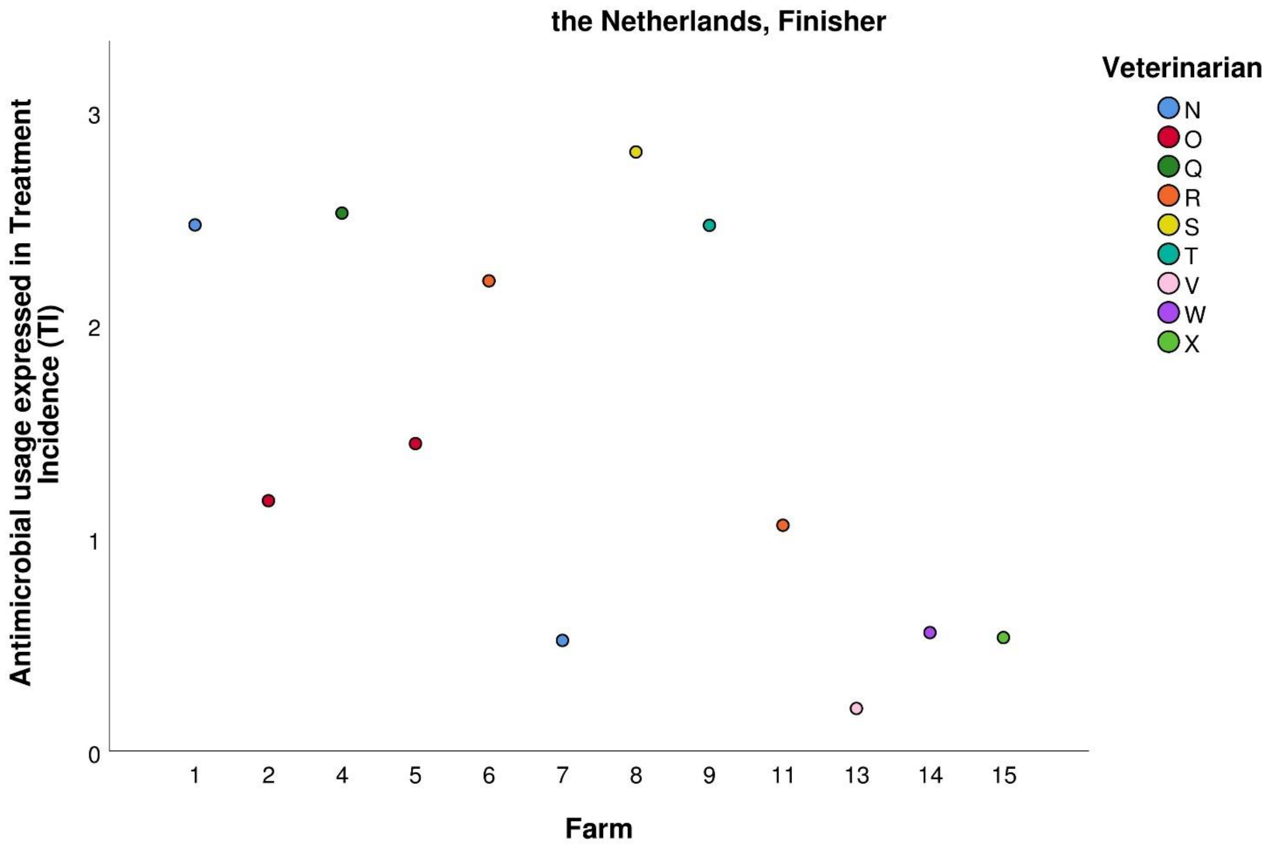

Supplementary Figure 8 Antimicrobial use of each participating pig farm in the Netherlands (n=15) for the animal category of the finishers. Antimicrobial use is expressed in Treatment Incidence (TI) on 100 days, the number of days an animal was treated with antimicrobials out of 100 days. The different colors represent the different veterinarians involved in this study. Farms 3, 10 and 12 had no finishers.
